# A Codon-Optimized Bacterial Antibiotic Gene Used as Selection Marker for Stable Nuclear Transformation in the Marine Red Alga ***Pyropia yezoensis***

**DOI:** 10.1007/s10126-013-9549-5

**Published:** 2013-10-23

**Authors:** Toshiki Uji, Ryo Hirata, Satoru Fukuda, Hiroyuki Mizuta, Naotsune Saga

**Affiliations:** Faculty of Fisheries Sciences, Hokkaido University, Hakodate, 041-8611 Japan

**Keywords:** *Pyropia yezoensis*, Red alga, Selection marker, Stable transformation

## Abstract

Marine macroalgae play an important role in marine coastal ecosystems and are widely used as sea vegetation foodstuffs and for industrial purposes. Therefore, there have been increased demands for useful species and varieties of these macroalgae. However, genetic transformation in macroalgae has not yet been established. We have developed a dominant selection marker for stable nuclear transformation in the red macroalga *Pyropia yezoensis*. We engineered the coding region of the aminoglycoside phosphotransferase gene *aph7″* from *Streptomyces hygroscopicus* to adapt codon usage of the nuclear genes of *P. yezoensis*. We designated this codon-optimized *aph7″* gene as *PyAph7*. After bombarding *P. yezoensis* cells with plasmids containing *PyAph7* under the control of their endogenous promoter, 1.9 thalli (or individuals) of hygromycin-resistant strains were isolated from a 10-mm square piece of the bombarded thallus. These transformants were stably maintained throughout the asexual life cycle. Stable expression of *PyAph7*was verified using Southern blot analysis and genomic PCR and RT-PCR analyses. *PyAph7* proved to be a new versatile tool for stable nuclear transformation in *P. yezoensis*.

**Electronic supplementary material:**

The online version of this article (doi:10.1007/s10126-013-9549-5) contains supplementary material, which is available to authorized users.

## Introduction

The marine red macroalga *Pyropia yezoensis* (*nori* in Japanese) is one of the most important marine crop. It is widely cultivated in eastern Asian countries, including Japan, Korea, and China, and generates US$1.3 billion per year (Blouin et al. 2011). In addition, *P. yezoensis* has attracted considerable interest as a model for physiological and genetic studies of marine red algae (Saga and Kitade 2002; Waaland et al. 2004). To date, several studies have been performed to make this alga a sophisticated model organism. For example, a laboratory culturing system in which the life cycle of *P. yezoensis* could be completed within a few months was established (Kuwano et al. 1996). In addition, a database for expressed sequenced tags (EST) analysis is now available (Nikaido et al. 2000; Asamizu et al. 2003) and recently, the draft data of whole genome sequence has been analyzed by next generation sequencing (Nakamura et al. 2013). However, a stable transformation system, a powerful tool both for elucidating gene functions and conferring valuable characteristics to an organism, has not yet been established for *P. yezoensis* or other marine macroalgae.

As an initial step in establishing stable transformation, we previously developed a transient gene expression system to monitor gene expression in *P. yezoensis* cells using particle bombardment. Because *P. yezoensis* genes have a strong GC bias in the third nucleotide of their codons, it is important to adapt codon usage of foreign genes to the nuclear genes of *P. yezoensis* for their efficient expression. In fact, codon-optimized β-glucuronidase (PyGUS) and GC-rich fluorescent proteins, such as AmCFP and sGFP(S65T), have been expressed in *P. yezoensis* cells under the control of an endogenous promoter (Fukuda et al. 2008; Mikami et al. 2009; Uji et al. 2010).

In addition to developing an efficient expression system, a reliable method to select and isolate transformed cells is required to establish stable transformation in macroalgae. Recently, we have revealed that *P. yezoensis* cells are sensitive to several aminoglycoside antibiotics, including hygromycin B, paromomycin, and geneticin (Takahashi et al. 2011). Thus, these antibiotics are possible candidate selection agents for stable *P. yezoensis* transformation.

Regarding a selection marker, the aminoglycoside phosphotransferase gene *aph7″* from *Streptomyces hygroscopicus*, which confers resistance against hygromycin B, should be available for hygromycin-based stable transformant selection because the GC content in its coding region is as high as 70.94 % (Zalacain et al. 1986). The *aph7″* gene has been successfully used for the transformation of green microalgae, such as *Chlamydomonas reinhardtii* (Berthold et al. 2002), whose codons are also rich in GC residues. However, several codons that are rarely used in *P. yezoensis* nuclear genes are found in the *aph7″* gene, especially in its N-terminal region. These mismatches in codon usage would be predicted to inhibit efficient translation in *P. yezoensis* cells.

Thus, in the present study, we synthesized a codon-optimized *aph7″* gene and examined its utility as a selection marker for stable nuclear transformation in *P. yezoensis*.

## Materials and Methods

### Culturing of *P. yezoensis*

Gametophytes of *P. yezoensis* strain TU-1 and transformants were cultured in enriched sea life (ESL) medium under conditions described by Fukuda et al. (2008).

### Plasmid Construction

To construct a pEA7 plasmid, a fragment containing the ORF of *PyAph7* and 3′ UTR of *CrRbcS2* was amplified using pHyg4 as a template and a pair of primers, XbaI-PyAph7-F (5′-GCTCTAGAATGACGCAGGAGTCCCTGCTGCTGCTC-3′) and *Eco*RI-CrRbcS2-R (5′-GGAATTCTTCCATGGGATGACGGGCCCGG-3′). The amplified PCR product was digested with *Xba*I and *Eco*RI and subsequently inserted into *Xba*I–*Eco*RI-digested p35S-PyGUS (Fukuda et al. 2008), which was designated p35S-PyAph7. To replace the CaMV 35S promoter with an endogenous promoter, the 5′ upstream region of *PyElf1* was amplified using pPyElf1-PyGUS (Mikami et al. 2011) as a template and the following primers: *Hin*dIII-PyElf1-F/XbaI-PyElf1-R (5′-CCCAAGCTTCCAGACCCGTGGAAAGTACCATC-3′/5′ GCTCTAGACTTGCCCATGGTGGGGGGG-3′). The PCR product was digested with *Hin*dIII and *Xba*I and subsequently inserted into the *Hin*dIII–*Xba*I site of p35S-PyAph7. This resulted in pEA7 construction.

### Particle Bombardment

Expression plasmids were purified from 100 mL of *Escherichia coli* culture using a NucleoBond Xtra Midi (MACHEREY-NAGEL, Germany). For particle bombardment, a gametophytic thallus with monosporangia covering a wide range of the thallus (>10 mm in width) was cut into 10-mm square pieces, which were subsequently placed on a filter paper. After removing excess fluid, the expression plasmids were introduced into the gametophytic cells using PDS-1000/He particle bombardment under the conditions described previously (Hirata et al. 2011).

### Isolation of Hygromycin-Resistant Transformants

The bombarded algal pieces were cultured in a 100-mL glass flask (Iwaki Sci Tech Div., Asahi Techno Glass, Japan) in 50 mL of ESL medium under non-selective conditions for 1 week. Subsequently, the medium was replaced with the ESL medium containing hygromycin B (final concentration of 1 mg mL^−1^), and the medium was renewed weekly. After incubation for 6–8 weeks in the antibiotic-containing medium, visible hygromycin-resistant transformants regenerated from the pieces of bombarded thalli were individually isolated in another culture flask and continuously cultured in ESL medium with or without 1 mg mL^−1^ hygromycin B.

### Assay for Hygromycin Resistance

To prepare individuals for hygromycin resistance assay, gametophytes isolated as hygromycin-resistant transformants were clonally propagated for 3 weeks in different culture flasks containing ESL medium via monospores. Gametophytes of transformants or wild-type strains (ca. 20 mm in length) cultured were respectively transferred into a 6-well plate (3 individuals/well) (Iwaki Sci Tech Div., Asahi Techno Glass, Japan) containing 5 mL of ESL medium with 0, 1.0, 2.5, 5.0, 7.5, or 10.0 mg mL^−1^ hygromycin B and incubated under shaking culture for 2 weeks at 15 °C. The medium was renewed weekly. After culture, gametophyte viability was estimated by staining using 0.01 % erythrosine (Wako Pure Chemical Industries, Japan) in ESL medium according to a previous report (Takahashi et al. 2011).

### Genomic PCR and RT-PCR

Genomic DNA was extracted from gametophytes of transformants cultured for 4 weeks in ESL medium without hygromycin B or wild-type strains for genomic PCR as described by Hwang et al. (2010) and purified using a phenol–chloroform extraction and ethanol precipitation. The precipitate was resuspended in 50 μL of TE buffer, and 2 μL of this suspension was used as a template for genomic PCR. RNA extraction and cDNA synthesis for RT-PCR were performed as described by Uji et al. (2012). Genomic PCR and RT-PCR analyses were conducted using *TaKaRa LA Taq* with GC buffer (TaKaRa-Bio). The primer pairs PyAph7-RT-F/R (5′-CATTGACTCGGACGACTCCTACGCGAG-3′/5′-AAGTCGTGCAGGAAGGTGAAG-3′) and PyElf1-RT-F/R (5′-AAGGCCAAGGCACCCAAGCTG-3′/5′-ACCACACCAAGAGCGTCCAATC-3′) were used to amplify the fragments of *PyAph7* (864 bp) and *PyElf1* (734 bp), respectively. The amplified PCR products were examined on a 1.3 % agarose gel.

### Southern Blotting

**Fig. 1:**
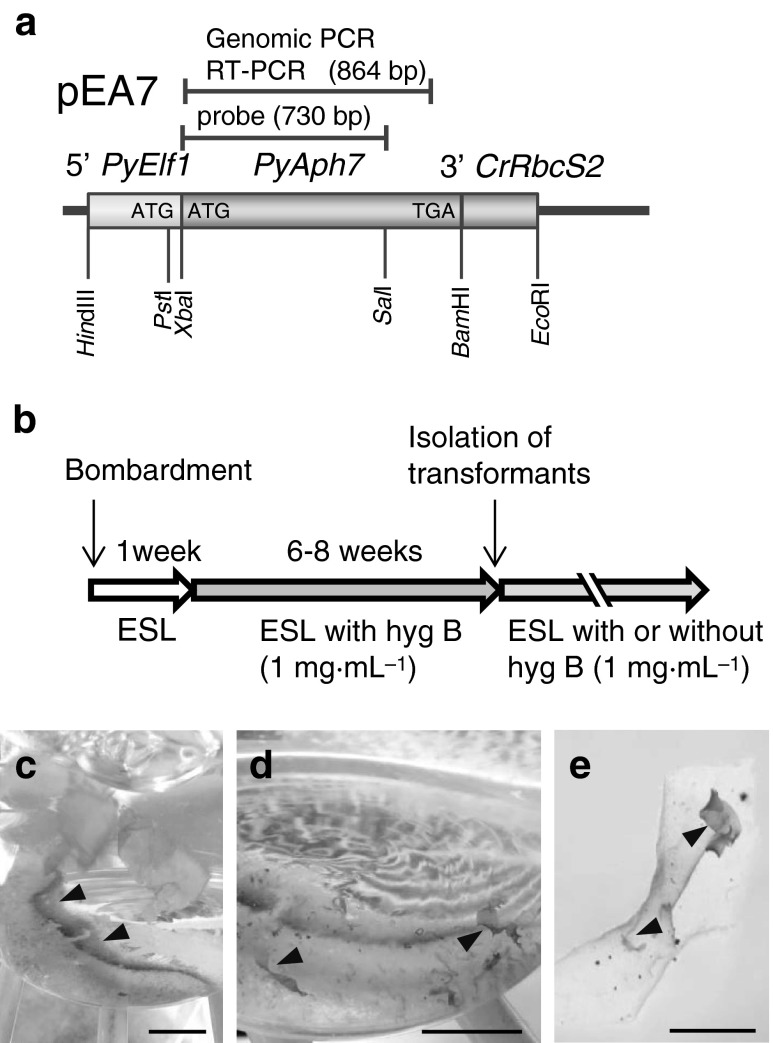
Isolation of hygromicin-resistant transformants in *P. yezoensis.* **a** Schematic diagram of the hygromycin selective vector pEA7. The coding region of *PyAph7* is fused in-frame to 5′ PyElf1 (promoter, 5′-untranslated region of *PyElf1* and the initiation codon). *3′ CrRbcS2* indicates the 3′ untranslated region of the *RbcS2* gene from *Chlamydomonas reinhardtii*. The position and length of the DNA fragment amplified by genomic PCR or RT-PCR are indicated. The position of the probe used in Southern blotting is indicated (probe). **b** Timeline for isolating hygromicin-resistant transformants (*hyg B*, hygromycin B). **c** Macroscopic view of the bottom of the culture flask on which monospores released from bombarded thalli were attached (*arrowheads*). Scale bar = 10 mm. **d** Hygromycin-resistant thalli regenerated from monospores attached to the bottom of a culture flask (*arrowheads*). Scale bar = 10 mm. **e** Hygromycin-resistant thalli regenerated from the vegetative cells of a bombarded thallus (*arrowheads*). Scale bar = 5 mm

Genomic DNA was extracted using the cetyl trimethyl ammonium bromide (CTAB) method from 1.0 g (FW) of wild type and transformants that had been cultured for more than 3 months after isolation. Extracted DNA was further purified by ultracentrifugation as follows: 3.1 g of cesium chloride (CsCl) and 15 μL of ethidium bromide (EtBr) were added to 3.0 mL of the DNA solution and centrifuged at 400,000 × *g* for 24 h at 20 °C. The DNA band was visualized under UV light and collected. EtBr was removed by three extractions with an equal volume of 1-butanol. CsCl was removed by ethanol precipitation three times. Purified DNA (2.0 μg) was digested with *Pst*I, run on agarose gel, and transferred to a nylon membrane. The pEA7 plasmid was digested with *Xba*I and *Sal*I and a 730-bp fragment of *PyAph7* was collected. This fragment was labelled by random 32P priming and used as a probe (see Fig. 1a).

## Results and Discussion

To optimize the codon usage of the *aph7″* coding region to that of *P*. *yezoensis*, we employed site-directed mutagenesis using a pHyg4 plasmid that contained the *aph7″* gene (Berthold et al. 2002) and a KOD-Plus-Mutagenesis Kit (Toyobo, Japan) with the oligonucleotides shown in Table S1. The synthetic *aph7″* gene was designated *PyAph7* (Fig. S1). Subsequently, the protein coding region of *PyAph7* was fused with the endogenous *PyElf1* promoter (Mikami et al. 2011). The resulting plasmid was designed pEA7 (Fig. 1a).

**Fig. 2:**
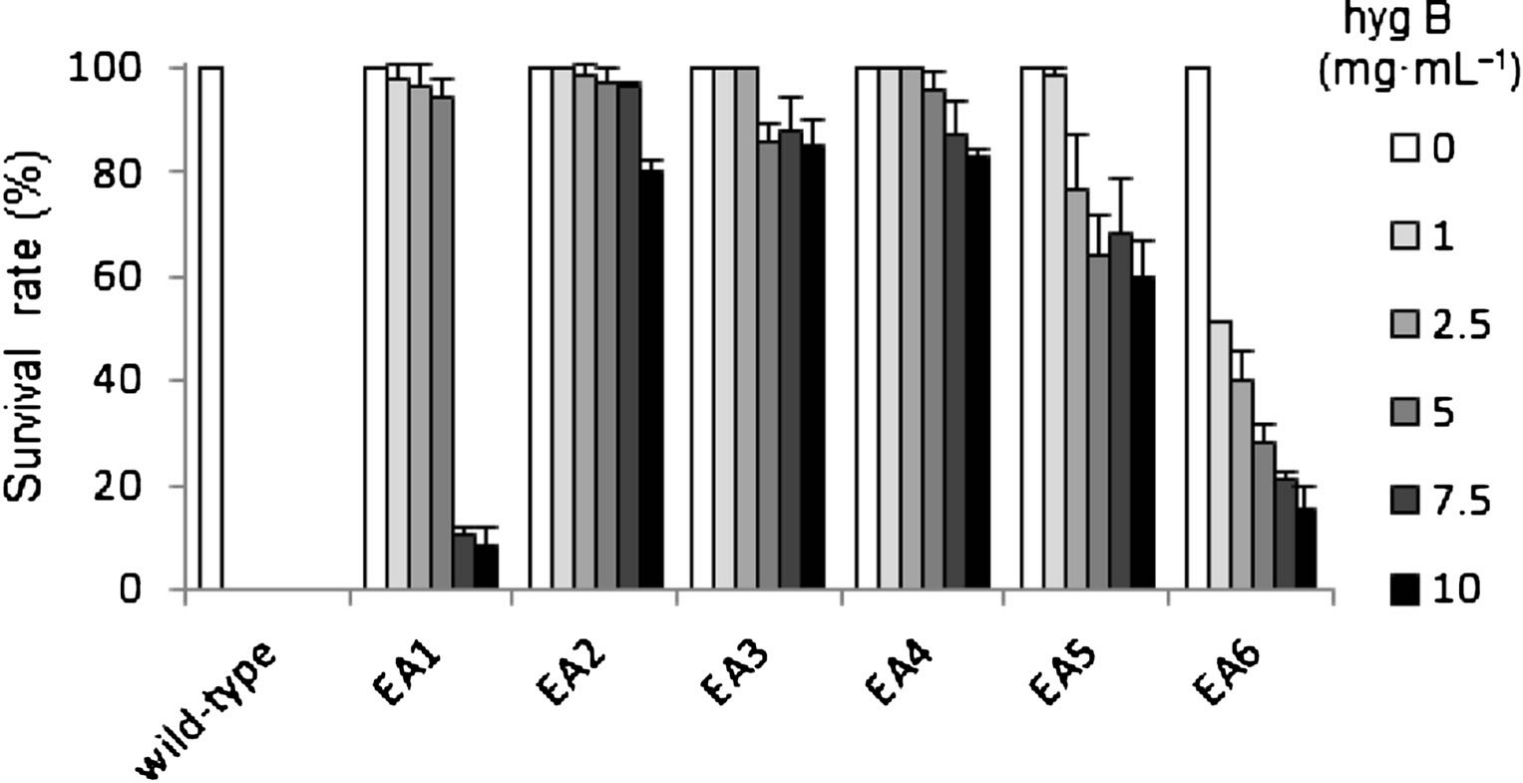
Analysis of hygromycin B resistance for wild-type and transgenic *P. yezoensis* strains. Survival rates of wild type and six lines of the hygromycin-resistant strains (EA1–EA6) when cultured with varying concentrations of hygromycin B (1.0–10.0 mg mL^−1^). The survival rate was calculated by counting viable and dead gametophytes during 2 weeks culture in ESL medium containing hygromycin B. Values are means ± SDs (*n* = 30)

The pEA7 expression plasmids containing *PyAph7* were introduced into *P*. *yezoensis* gametophytes by particle bombardment. When gametophytic thalli with the introduced pEA7 were cultured in a non-selective ESL medium for 1 week (Fig. 1b), some monospores (asexual spores) were released from the bombarded thalli and adhered to the bottom of the culture flask (Fig. 1c). The medium was subsequently replaced with selective ESL medium containing 1.0 mg mL^−1^ hygromycin B, which effectively kills wild-type cells or, at least, completely inhibits their growth (Takahashi et al. 2011). After 6–8 weeks of culture in the selective medium, hygromycin-resistant thalli from them released monospores or from vegetative cells of bombarded blades were approximately 5–10 mm long (Fig. 1d, e). These transformants were transferred into separate culture vessels to identify the homogeneous lines and cultured further in a non-selective ESL medium (Fig 1b). Consequently, an average of 1.9 thalli (or individuals) of the hygromycin-resistant strains were obtained from a piece of the bombarded thallus (29 transformants per 15 bombardments). These transformants were successfully maintained over more than five generations as independent lines through the asexual life cycle via monospores. Subsequently, we examined the hygromycin B tolerance of six isolated transformants: EA1–EA6. As shown in Figs. 2 and S2, when the gametophytes were cultured in an ESL medium containing 1.0 mg mL^−1^ hygromycin B, all wild-type gametophytes were dead after 2 weeks of culture. In contrast, all transformants survived and grew in this medium. Transformants EA2, EA3, and EA4 survived even in the presence of 2.5–10.0 mg mL^−1^ hygromycin B (Figs. 2 and S2).

**Fig. 3:**
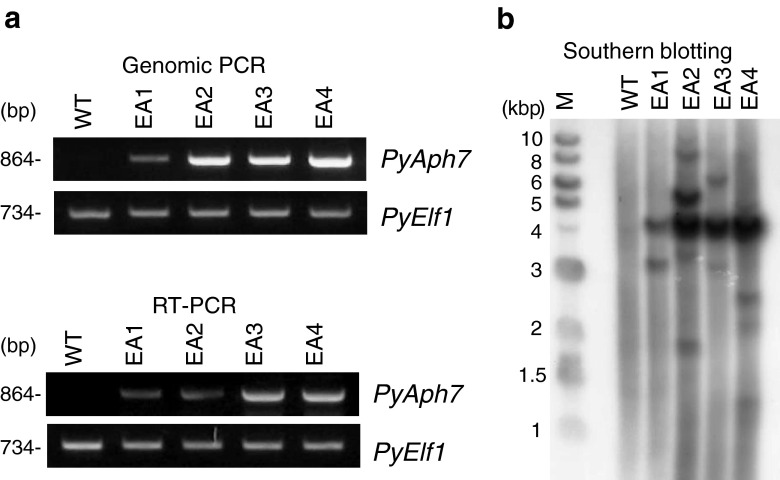
PCR and Southern blot analyses of hygromicin-resistant transformants. **a** Expression of the exogenous *PyAph7* gene in hygromycin-resistant transformants was detected by genomic PCR and RT-PCR. PCR was performed using primers specific for the *PyAph7* gene sequence (Fig. 1) and genomic DNA or total RNA from a wild-type strain and the transformants EA1–EA4. *PyElf1* was used as the internal control gene in *P. yezoensis*. Only transformants were expected to yield an 864 bp fragment of *PyAph7*. **b** Southern blot analysis of hygromycin-resistant transformants. Genomic DNA from a wild-type strain and the transformants EA1–EA4 were digested with *Pst*I, separated on agarose gel, transferred to nylon membrane, and hybridized with a labelled probe corresponding to the *PyAph7* fragment (Fig. 1). *Lane M*, molecular weight marker

Finally, to verify whether the exogenous *PyAph7* gene had been successfully introduced and expressed in these isolated transformants, genomic PCR and RT-PCR analyses were performed on four transformant strains (EA1–EA4). Using primers specific for the *PyAph7* gene sequence, a DNA fragment of the expected size was amplified for all of the examined strains, whereas this fragment was not observed in a wild-type strain (Fig. 3a). This indicated that *PyAph7* had been successfully introduced and expressed in these transformants. We further analyzed the stable integration of *PyAph7* into the genome by Southern blot analysis. The Southern blot analysis revealed that multiple DNA fragments could be detected in all of the examined transformants, which indicated that *PyAph7* had multiplied and randomly integrated into the genome (Fig. 3b). Strong signals of an approximately 4.2 kbp DNA fragment that corresponded to the full length of the pEA7 vector were commonly detected in all transformed strains. The results intimate the possibility that a part of introduced pEA7 vector might be stably maintained as entire circular plasmids in the transformed cells through cell divisions and propagation. Interestingly, there have been several reports on plasmid DNA isolation from some red macroalgae, including *Pyropia tenera*, a closely related species of *P. yezoensis* (Goff and Coleman 1990; Choi et al. 2000, 2001). Thus, we need to investigate on mechanisms for maintaining plasmid DNA in *P. yezoensis* cells.

Taken together, the codon-optimized *PyAph7* introduced by particle bombardment was stably maintained and expressed in *P. yezoensis* and conferred resistance to hygromycin B. Our results demonstrate that *PyAph7* is useful as an efficient selection marker for stable nuclear transformation of *P. yezoensis*. This is the first report of a bacterial antibiotic gene used as a selection marker for stable transformation in marine macroalgae. Further development of this stable transformation system in *P. yezoensis* will overcome some of the barriers in molecular biological studies of marine red algae.

## Electronic supplementary material

Below is the link to the electronic supplementary material.

Table S1 Oligonucleotides used for artificial synthesis of *PyAph*7 gene (DOC 69 kb)

ESM 2 (DOC 63 kb)

ESM 3 (DOC 647 kb)

## References

[CR1] Asamizu E, Nakajima M, Kitade Y, Saga N, Nakamura Y, Tabata S (2003). Comparison of RNA expression profiles between the two generations of *Porphyra yezoensis* (Rhodophyta), based on expressed sequence tag frequency analysis. J Phycol.

[CR2] Berthold P, Schmitt R, Mages W (2002). An engineered *Streptomyces hygroscopicus aph 7*″ gene mediates dominant resistance against hygromycin B in *Chlamydomonas reinhardtii*. Protist.

[CR3] Blouin NA, Brodie JA, Grossman AC, Xu P, Brawley SH (2011). *Porphyra*: a marine crop shaped by stress. Trends Plant Sci.

[CR4] Choi HS, Choi KH, Rhew TH (2000). Simple and rapid isolation of plasmids from *Porphyra tenera*. Algae.

[CR5] Choi HS, Choi KH, Kim TH, Lee CH, Rhew TH (2001). Characterization of natural plasmid and construction of putative transformation vector using the plasmid in Korean red alga, *Porphyra tenera*. Algae.

[CR6] Fukuda S, Mikami K, Uji T, Park EJ, Ohba T, Asada K, Kitade Y, Endo H, Kato I, Saga N (2008). Factors influencing efficiency of transient gene expression in the red macrophyte *Porphyra yezoensis*. Plant Sci.

[CR7] Goff LJ, Coleman AW (1990). Red algal plasmids. Curr Genet.

[CR8] Hwang BK, Son SH, Lee JS, Min SR, Ko SM, Liu JR, Choi DS, Jeong WJ (2010). Rapid and simple method for DNA extraction from plant and algal species suitable for PCR amplification using a chelating resin Chelex 100. Plant Biotechnol Rep.

[CR9] Hirata R, Takahashi M, Saga N, Mikami K (2011). Transient gene expression system established in *Porphyra yezoensis* is widely applicable in Bangiophycean algae. Marine Biotechnol.

[CR10] Kuwano K, Aruga Y, Saga N (1996). Cryopreservation of clonal gametophytic thalli of *Porphyra* (Rhodophyta). Plant Sci.

[CR11] Mikami K, Uji T, Li L, Takahashi M, Yasui H, Saga N (2009). Visualization of phosphoinositides via the development of the transient expression system of a cyan fluorescent protein in the red alga *Porphyra yezoensis*. Marine Biotechnol.

[CR12] Mikami K, Hirata R, Takahashi M, Uji T, Saga N (2011) Transient transformation of red algal cells: breakthrough toward genetic transformation of marine crop *Porphyra* species. In María Alvarez (ed) Genetic transformation*.* InTech Open Access, p 241–258

[CR13] Nakamura Y, Sasaki N, Kobayashi M, Ojima N, Yasuike M, Shigenobu Y, Satomi M, Fukuma Y, Shiwaku K, Tsujimoto A, Kobayashi T, Nakayama I, Ito F, Nakajima K, Sano M, Wada T, Kuhara S, Inouye K, Gojobori T, Ikeo K (2013). The first symbiont-free genome sequence of marine red alga, susabi-nori (*Pyropia yezoensis*). PLoS One.

[CR14] Nikaido I, Asamizu E, Nakajima M, Nakamura Y, Saga N, Tabata S (2000). Generation of 10,154 expressed sequence tags from a leafy gametophyte of a marine red alga, *Porphyra yezoensis*. DNA Res.

[CR15] Saga N, Kitade Y (2002). *Porphyra*: a model plant in marine sciences. Fish Sci.

[CR16] Takahashi M, Mikami K, Mizuta H, Saga N (2011). Identification and efficient utilization of antibiotics for the development of a stable transformation system in *Porphyra yezoensis* (Bangiales, Rhodophyta). J Aquac Res Development.

[CR17] Uji T, Takahashi M, Saga N, Mikami K (2010). Visualization of nuclear localization of transcription factors with cyan and green fluorescent proteins in the red alga *Porphyra yezoensis*. Marine Biotechnol.

[CR18] Uji T, Hirata R, Mikami K, Mizuta H, Saga N (2012). Molecular characterization and expression analysis of sodium pump genes in the marine red alga *Porphyra yezoensis*. Mol Biol Rep.

[CR19] Waaland JR, Stiller JW, Cheney DP (2004). Macroalgal candidates for genomics. J Phycol.

[CR20] Zalacain M, Gonzalez A, Guerrero MC, Mattaliano RJ, Malpartida F, Jimenez A (1986). Nucleotide sequence of the hygromycin B phosphotransferase gene from *Streptomyces hygroscopicus*. Nucleic Acids Res.

