# Supplementary figures and images for: A Codon-Optimized Bacterial Antibiotic Gene Used as Selection Marker for Stable Nuclear Transformation in the Marine Red Alga Pyropia yezoensis
Source: Mar Biotechnol (NY). 2013 Oct 23;16(3):251–5. doi: 10.1007/s10126-013-9549-5 (PMC3996358; doi:10.1007/s10126-013-9549-5)

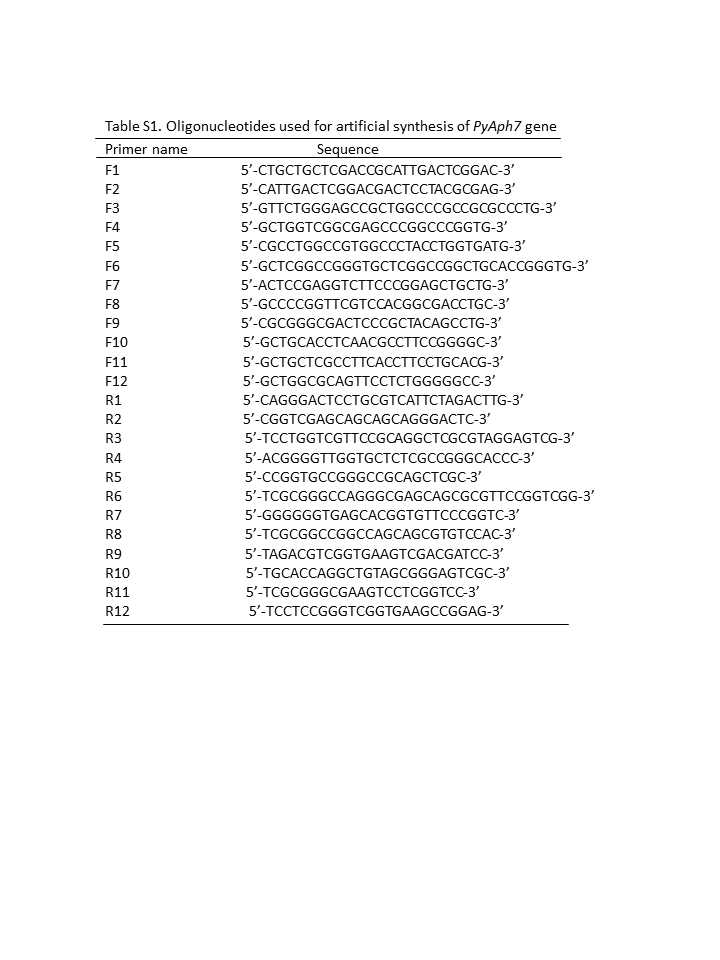

Supplement: Supplementary file 1 — Oligonucleotides used for artificial synthesis of PyAph7 gene (DOC 69 kb) [file 10126_2013_9549_MOESM1_ESM.doc]

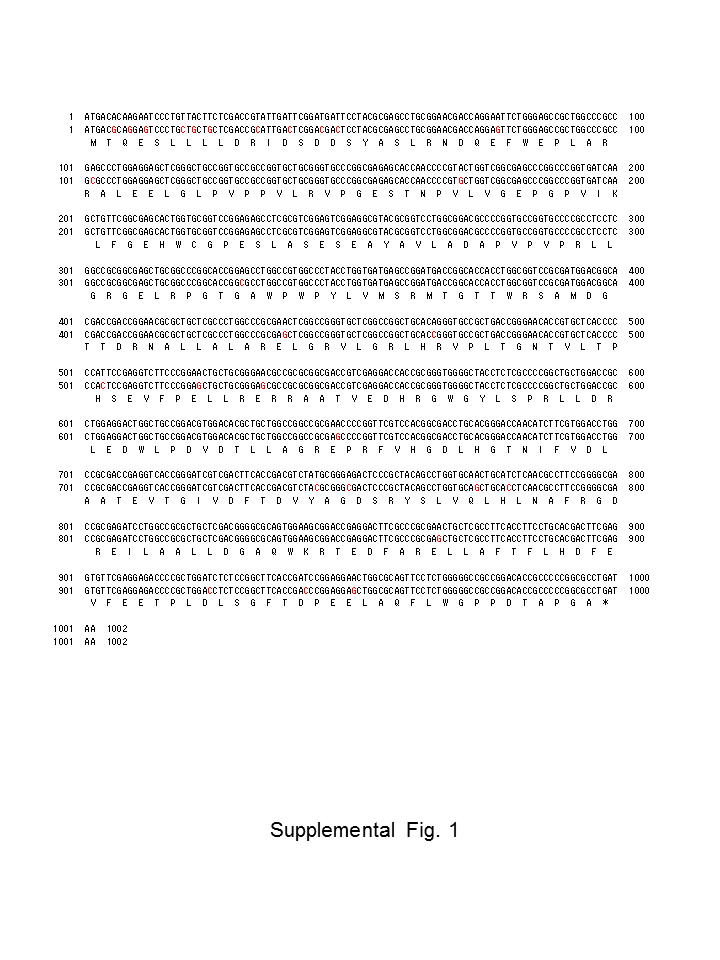

Supplement: Supplementary file 2 — (DOC 63 kb) [file 10126_2013_9549_MOESM2_ESM.doc]

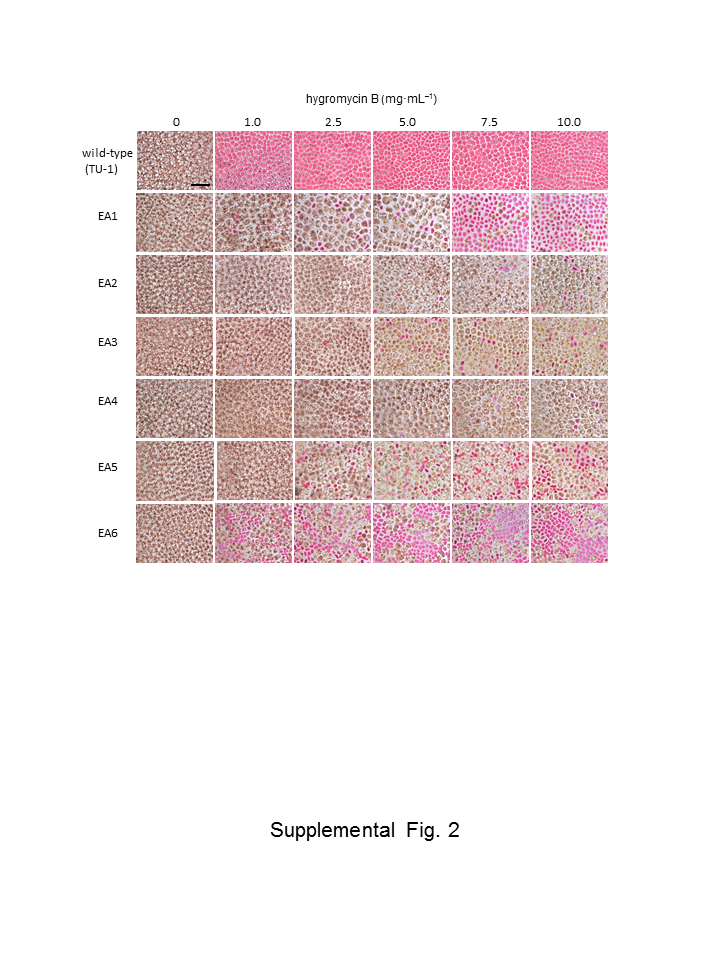

Supplement: Supplementary file 3 — (DOC 647 kb) [file 10126_2013_9549_MOESM3_ESM.doc]
